# New Insights from the High-Resolution Monitoring of Microalgae–Virus Infection Dynamics

**DOI:** 10.3390/v14030466

**Published:** 2022-02-24

**Authors:** Gino Schiano di Visconte, Michael J. Allen, Andrew Spicer

**Affiliations:** 1 Algenuity Limited, Eden Laboratory, Broadmead Road, Stewartby MK43 9ND, UK; andrew.spicer@algenuity.com; 2 Streatham Campus, College of Life and Environmental Sciences, University of Exeter, Exeter EX4 4QD, UK; m.allen5@exeter.ac.uk; 3 Plymouth Marine Laboratory, Plymouth PL1 3DH, UK

**Keywords:** microalgae, biotechnology, *Chlorella*, virus, photobioreactor

## Abstract

Investigation of virus-induced microalgal host lysis and the associated infection dynamics typically requires sampling of infected cultures at multiple timepoints, visually monitoring the state of infected cells, or determining virus titration within the culture media. Such approaches require intensive effort and are prone to low sensitivity and high error rates. Furthermore, natural physiological variations can become magnified by poor environmental control, which is often compounded by variability in virus stock efficacy and relatively long infection cycles. We introduce a new method that closely monitors host health and integrity to learn about the infection strategy of Chloroviruses. Our approach combines aspects of spectrometry, plaque assays, and infection dose assessment to monitor algal cells under conditions more representative of the natural environment. Our automated method exploits the continuous monitoring of infected microalgae cultures in highly controlled lab-scale photobioreactors that provide the opportunity for environmental control, technical replication, and intensive culture monitoring without external intervention or culture disruption. This approach has enabled the development of a protocol to investigate molecular signalling impacting the virus life cycle and particle release, accurate determination of virus lysis time under multiple environmental conditions, and assessment of the functional diversity of multiple virus isolates.

## 1. Introduction

There are a variety of tools, techniques, and methodologies for characterising viruses, their life cycles, and their properties. Broadly speaking, three main approaches are taken: direct observations derived from their impact on their host (such as viral plaque assay [1], infection dose (ID_50_) [2], and immunofluorescence foci assays [3]); observations based upon specific components of the virus or infected hosts (e.g., quantification of viral nucleic acid and protein, including qPCR [4], immunoblotting [5], immunoprecipitation [6], ELISA [7], mass spectrometry [8], and next-generation sequencing/metagenomics [9,10]); and, lastly, direct physical assessment/observation of whole viral particles (via flow cytometry [11], scanning/transmission electron microscopy [12], or confocal microscopy [13]).

For the microalgal viruses, the most commonly used techniques are viral plaque assay [14], transmission electron microscopy [15], and flow cytometry [16], together with mass spectrometry for metabolite analysis and next-generation sequencing/metagenomics for genomic/transcriptomic analysis. Few generically applicable protocols have been developed that exploit monitoring of the physiological state of the host cells to learn about the presence and activity of these poorly understood yet fascinating viruses.

Previously, in order to determine Phycodnavirus host lysis and/or release time, the only possible way was to sample infected cultures at multiple timepoints and monitor visually the state of the individual infected cells or determine a virus particle count in the media. Such approaches require intensive effort and are prone to low sensitivities. They are further exacerbated by poor environmental control, variation in virus stock quality, and relatively long infection life cycles.

Here, we introduce a new method that closely monitors host health and integrity to gain new insights regarding the infection strategy of the *Chlorella variabilis* infecting Chloroviruses. Our automated method exploits the continuous monitoring of infected microalgae cultures in highly controllable lab-scale photobioreactors (PBR) that provide the opportunity for environmental control, technical replication, and intensive monitoring, crucially, without a requirement for external intervention or disruption. In conjunction with established techniques, this novel application of PBR technology enabled the optimisation of growth conditions and medium composition, the identification of subtle differences in virus life cycle and multiplicity of infections (MOI), a more accurate estimation of plaque forming units (pfu) released per lysed cell, the exploration of virus release strategies, as well as the investigation of the role of potential signalling molecules in controlling the virus life cycle.

## 2. Materials and Methods

### 2.1. Chemicals

Culture media components were purchased from VWR international, UK. All other chemicals were purchased from Merck, UK.

### 2.2. Microalgae Strain, Virus Isolates, and Growth Conditions

*Chlorella variabilis* CCAP 211/84 was obtained from the Culture Collection of Algae and Protozoa (CCAP) located within the Scottish Association for Marine Science campus (SAMS) [17]. The strain was maintained on Tris-acetate-phosphate (TAP) medium [18] with added vitamins (vitamin B1 1 mg/L final concentration and B12 12 μg/L final concentration) on 1.5% agar plates and re-streaked every month. Three media, prepared as per reference, were tested: TAP medium [18] with added vitamins, high salt medium (HSM) with added vitamins [19], and Bold’s basal medium (BBM) [20] modified by the addition of 0.5% sucrose and 0.1% proteose-peptone (MBBM) [14]. The carbon sources tested in HSM were all at the same concentration of 3%. The nitrogen sources tested in TAP medium were normalised to the number of nitrogen atoms in the media.

Virus isolates PBCV-1, CviKI, IL-5-2S1, NYs-1, and MA-1D were gifted by Prof. David D. Dunigan’s laboratory. Virus isolate TAAS 2.1 was one of a number of viral isolates obtained from water samples taken from small ponds located in rural New Hampshire, USA using viral plaque assay [14] using TAP medium plates with added vitamins. Fresh viral stocks were prepared infecting *Chlorella variabilis* cultures in exponential phase (5-10 × 10^6^ cells/mL), and collecting the lysed culture 24 h post-infection (p.i.); viral lysates were stored at 4 °C.

Titration of the viral stocks was performed in triplicate using viral plaque assays of serial dilutions of the supernatant of lysed cells, as per reference [14], using TAP agar plates with added vitamins as an alternate and richer medium. By using TAP medium, *Chlorella* monolayers growing within the soft top agar greened significantly faster as compared to the more conventional published and widely used approach that uses MBBM [14]. Results were expressed as average ± standard deviation in pfu/mL.

TAP medium [18] with added vitamins was also used for liquid cultures. Cultures were grown in Algem^®^ HT24 photobioreactor [21] in 25 mL volume inside 50 mL clear and clean glass Pyrex Erlenmeyer flasks or Algem PRO photobioreactor [21] in 400 mL volume in 1 L clear and clean Pyrex Erlenmeyer flasks. In both cases, the growth conditions were 28 °C with white LED light intensity set at 200 micromoles/m^2^/s (µM/m^2^/s micro-Einsteins (µE)) photosynthetically active radiation (PAR), measuring the OD at 740 nm every 10 min. The mixing was set to 120 rpm and gas aeration within the Algem PRO cultures was set with a flow rate of 25 mL/min of 30% CO_2_/air in order to control the pH of the culture at pH 7 via computer-controlled solenoid valve. All experiments were performed in triplicate. The average growth rate of the medium optimisation experiment, was calculated between day 1 and day 2 of cultivation by subtracting the OD value at day 1 from the OD value at day 2 and, because it was calculated over exactly 1 day, the value is divided by 1. Dry weight (DW) was measured by centrifuging 20 mL of culture, washing the pellet with 1 mL of deionised water once, and the pellet was left at 70 °C overnight with the tube open to dry. Cell count was performed manually using Olympus BH2 microscope with phase-contrast 10x magnification lens, using a cell count chamber (VWR international, UK).

The culture crash times were estimated by calculating the first derivatives of all curves and, based on the data collected from PBCV-1 lysis, the value of −0.006 of the first derivative, which shows the moment at which the curve has a significantly faster decrease in the OD, was determined to be the start of the culture crash time. The lysis time for all other virus isolates was calculated based on this assumption and verified with microscopy monitoring where lysis times were feasible to do so. The average lysis times and the standard deviations were calculated based on 3 independent biological replicates.

To calculate the order of magnitude of the number of virus particles released per cells, the following formula was used considering values of MOIs greater than 1 as MOI 1, assuming in the case of multiple infections per cell that overall virus production capacity is identical:

(1) $$10^{n}\;\left(\frac{pfu}{cell}\right)=\sqrt[{\frac{\left(average\;culture\;crash\;time\;of\;MOI=1\right)}{(average\;culture\;crash\;time\;of\;MOI<1)-\left(average\;culture\;crash\;time\;of\;MOI=1\right)}}]{10^{-\log_{10}(MOI<1)}}$$

For more accurate calculations, the experiment was repeated with multiple MOIs < 1 and the results were plotted as culture crash time in hours (y axis) against the log_10_ of the MOI, again considering values of MOIs greater than 1 as MOI 1. The linear trendline between the points was derived and the calculated values using the formula from the trendline were used to calculate the order of magnitude of the number of virus particles released per cell with the formula mentioned earlier.

For the verification of the presence of signalling molecules, spent medium was collected at 7 h p.i. from cultures infected with an MOI of 5, 10^−4^, and 10^−6^ and filtered through a 0.2 μm filter (Minisart NML Plus hydrophilic, GF+Cellulose acetate, VWR international, UK) initially and a 10 kDa MW cut-off PES filter (Vivaspin 20 Centrifugal concentrator, VWR international, UK) afterwards, and used to resuspend the cells prior to the infection at an MOI of 10^−4^.

The virus release method was verified by infecting three 25 mL cultures of *Chlorella variabilis* in TAP with added vitamins in the exponential phase with an MOI of 5. One hour p.i., the cells were harvested via centrifugation at 6000× *g* for 2 min, resuspended in 1 mL fresh TAP medium, and moved into a 2mL tube. Cells were harvested again via centrifugation at 6000× *g* for 2 min and resuspended again in 1 mL of fresh TAP medium. This process was repeated 5 times and was used to remove all unbound virus particles or loosely bound particles. At the end of the 5 washes, the cells were resuspended in 25 mL fresh TAP medium, and 1 mL was harvested every hour for the first 7 h p.i. and after 24 h p.i. via centrifugation at 6000× *g* for 2 min, and filtered with a 0.45 μm filter (Minisart NML hydrophilic, Cellulose acetate, VWR international, UK) to eliminate any residual infected cells. A total of 0.5 mL of each was used for the infection of a 25 mL culture and monitored in the Algem HT24.

## 3. Results

### 3.1. Growth Medium Optimisation

The reported standard growth conditions for *Chlorella variabilis* are well established [14,22] and, since 1965, the same medium (MBBM) has been utilised predominantly, despite supporting slow growth and low maximum culture cell densities at stationary phase.

**Figure 1:**
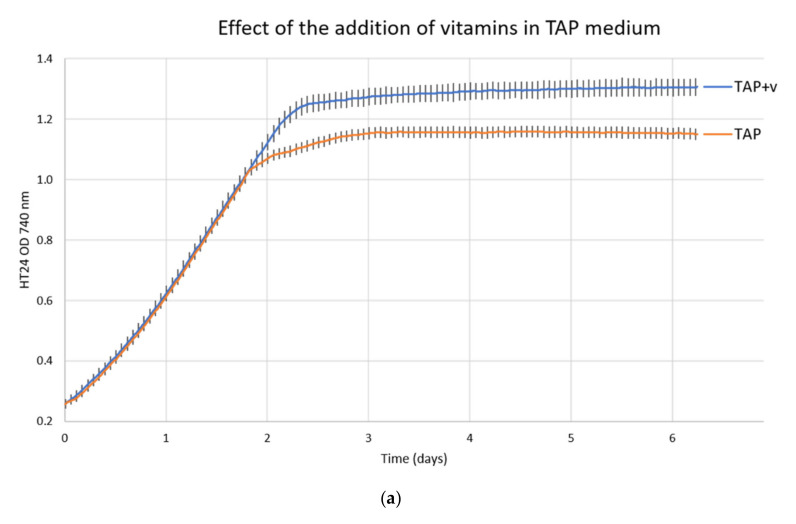

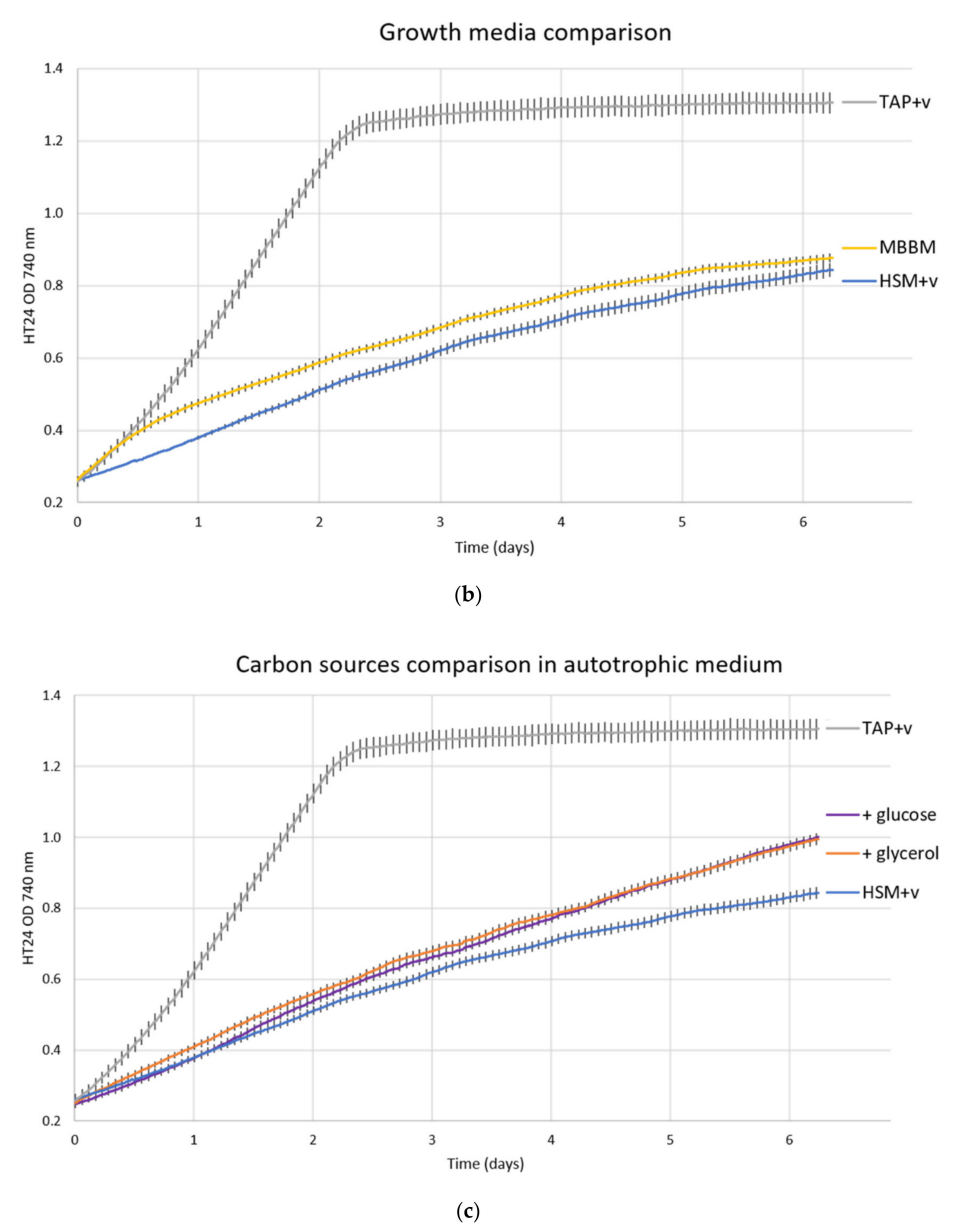

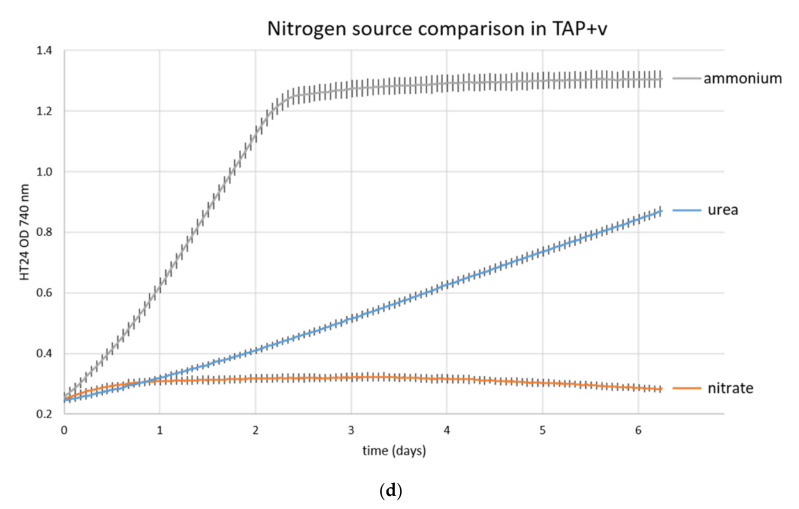
Growth of *Chlorella variabilis* in different media compositions. (**a**) Comparison of growth in TAP and TAP with added vitamins (TAP+v); (**b**) comparison of growth in HSM+v, TAP+v, and MBBM; (**c**) impact of carbon sources in HSM+v medium in comparison with TAP+v medium; (**d**) effect of nitrogen sources on cell growth in TAP+v medium. The plotted values are the average of 3 biological replicates with standard deviations.

Two additional media (TAP and HSM) identified from the literature have been tested to assess their suitability to support the growth of this strain, TAP and HSM, but, after subculturing *Chlorella variabilis* in these media several times, growth was observed to gradually decrease. Through inclusion/exclusion experiments, we subsequently identified that the presence of vitamins B1 and B12 are essential for the growth of this microalgal strain. In Figure 1a, the comparison between TAP and TAP+v, sub-cultured for the first time from TAP+v, is shown.

The growth rate is identical, but it reached stationary phase at a lower cell density. Presumably, in the MBBM medium, they are provided by the proteose-peptone fraction, which also provides nitrogen in the form of amino acids, making the investigation of nitrogen source and optimal content challenging.

The comparison of TAP and HSM, both with added vitamins with MBBM, presented in Figure 1b, shows that the mixotrophic condition with acetate, represented by the TAP medium, yields the highest growth rate (Table 1) and final biomass density.

To benchmark against the acetate found in TAP medium, alternative carbon sources were investigated in HSM with added vitamins (HSM+v): glucose, fructose, galactose, maltose, arabinose, lactose, sucrose, and glycerol were investigated. Fructose, galactose, maltose, arabinose, lactose, and sucrose had a negligible impact on growth rate or final biomass. However, glucose and glycerol had a positive impact on growth rate and final biomass density achieved, but were inferior to the acetate-based TAP+v media, as shown in Figure 1c. Utilising TAP+v medium variants, we investigated the impact of three different nitrogen sources: ammonium chloride (as in the original recipe), urea, and potassium nitrate on growth (Figure 1d). The strongest growth was obtained using ammonium, but the strain was also able to process urea; however, no growth was observed on nitrate, which is the nitrogen source in the original BBM, supporting the notion that proteose-peptone is providing the nitrogen necessary for the growth of this strain when in MBBM. The optimal media was found to be TAP with added vitamins using ammonium chloride as the nitrogen source, which yielded more than 3.5 times more biomass than MBBM after 6 days, and the growth rate was more than four times higher than that observed in MBBM. We used TAP+v media with ammonium chloride in all the subsequent infection experiments.

### 3.2. Viral-Induced Lysis at Saturating MOI

**Figure 2:**
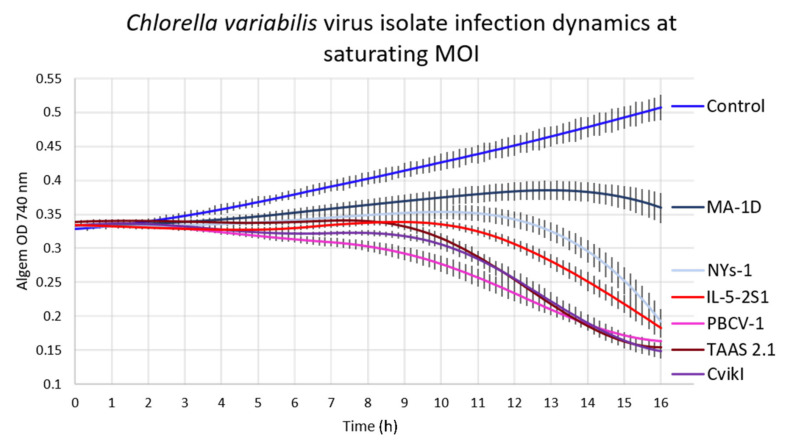
Infection of *Chlorella variabilis* cultures at an MOI of 5 with PBCV-1, MA-1D, NYs-1, TAAS 2.1, CviKI, and IL-5-2S1 virus isolates and uninfected control cultures of *Chlorella variabilis* strain CCAP 211/84. The plotted values are the average of 3 biological replicates with standard deviations.

*Chlorella variabilis* cells were cultured in TAP medium with added vitamins in Algem PRO photobioreactors and maintained in exponential growth phase, subculturing them weekly starting at 10^5^ cells/mL. Cells were infected in triplicate with a multiplicity of infection (MOI) of 5, i.e., 5 pfu per cell, for each of the different virus isolates. The optical densities at 740 nm of the infected cultures were automatically measured every 10 min for 16 h. As shown in Figure 2 and reported in the literature [23], following infection, the cells stopped growing compared to the noninfected cultures, for which the OD increases rapidly.

At differing times p.i., relative to each strain isolate, the measured OD values of infected cultures began to decrease as the cultures started crashing. All virus isolates caused a unique and reproducible absorbance profile in the host algal strain, reflecting and revealing differences in their life cycle dynamic, including average lytic cycle length. To rule out any impact due to potential variability in quality of viral stocks, we performed similar experiments changing the MOI to 10^−4^, namely, 1 pfu per 10^4^ cells. Culture crash times at lower MOI (10^−4^ MOI) were used to gather data about the number of pfu released per cell, reasoning that the more pfu released per cell, the shorter the difference between the lysis times at the different MOIs will be. The choice of 10^−4^ MOI is based on previous knowledge from PBCV-1 virus that releases about 200 pfu per lysed cell [23]. Under this scenario and assuming complete mixing of cultures, PBCV-1 would infect all cells in the culture within two viral lytic cycles and complete culture crash would be achieved within three lytic cycles.

### 3.3. Culture Crash Time Calculations and Estimation of Virus Particle Release per Cell at Lysis

**Figure 3:**
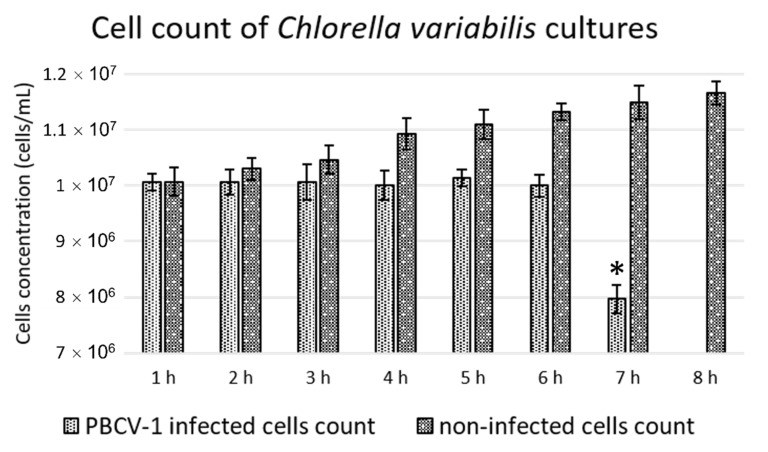
Cell count of PBCV-1-infected (lighter bar) and noninfected (darker bar) *Chlorella variabilis* cultures for the estimation of the culture crash time. The data represent the average of 3 biological replicates with calculated standard deviations. The asterisk indicates statistical significance compared to the previous timepoints with a *p*-value lower than 0.01.

Three saturating MOI PBCV-1-infected cultures were monitored every hour with manual cell count using the microscope, up to 8 h p.i., to determine when the cells started lysing. As shown in Figure 3, before 7 h p.i., there was no significant difference in the relative host cell density (10^7^ cells/mL).

**Figure 4:**
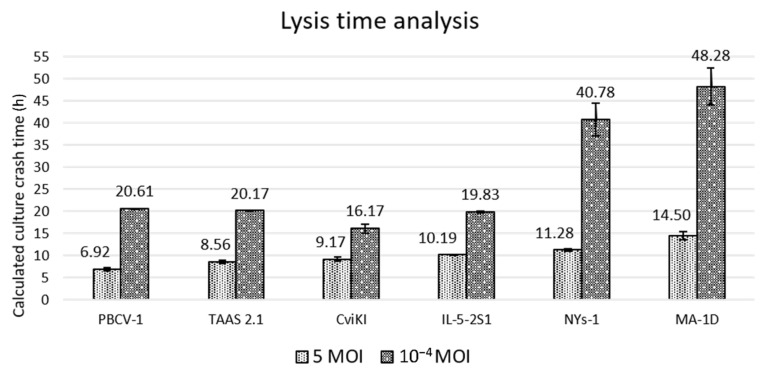
Calculated lysis time for PBCV-1, MA-1D, NYs-1, TAAS 2.1, CviKI, and IL-5-2S1 virus isolates. The data represent the average of 3 biological replicates with calculated standard deviations.

At 7 h p.i., the culture density was lower and statistically significant when compared against previous time points of the infected culture, with a p-value lower than 0.01. After 8 h p.i., most of the cells were lysed. Based on this result, we infer that the PBCV-1-infected CCAP 211/84 cells started to lyse after 6 h p.i. under the conditions tested. The culture crash time was calculated for all conditions, as mentioned in the material and methods section and reported in Figure 4.

CviKI has the lowest ratio between culture crash time at the different MOIs compared to the other viruses included in this analysis, suggesting a greater stability of virus particles, faster lytic cycle at lower MOIs, or largest burst size (number of virus particles released per single cell). Therefore, as the most robust and reliable virus system, CviKI was chosen to test further possible applications of the Algem HT24 high-throughput photobioreactor.

A novel approach may be used to calculate the order of magnitude of the pfu/cell for all viral isolates. Some viruses might have a greater binding capacity to cell debris, in which case the measurement of pfu/cell using plaque assay in the spent media would give an underestimation of the true value. The results of the calculation for PBCV-1, for which the value is known and reported [23], and for CviKI are reported and compared to the values determined by plaque assays. In this instance, for PBCV-1, the two values coincided: (200 ± 10) pfu/cell from the plaque assay and 10^2^ as the order of magnitude calculated using the culture crash times at the two different MOIs. For CviKI, the two values were different by two orders of magnitude: (1000 ± 200) pfu/cell measured with the plaque assay and a calculated order of magnitude of 10^5^ pfu/cell. For confirmation of the calculated value based on the culture crash time observed, we infected the microalgae with CviKI virus at multiple MOIs.

### 3.4. Analysis of Observed Culture Crash Time for CviKI Infection Based on MOI

**Figure 5:**
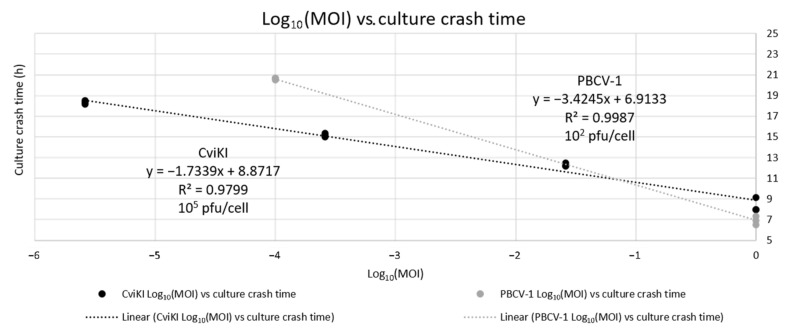
Linear regression of Log_10_ of the MOIs versus the culture crash time of infected cells with PBCV-1 and CviKI viruses. The data are reported as three biological replicates for each condition. The linear trendlines were calculated using excel and the R^2^ values are reported.

Microalgae cultures were infected with CviKI virus in triplicate at saturating MOI, 2.6 × 10^−2^ MOI, 2.6 × 10^−4^ MOI, and 2.6 × 10^−6^ MOI and monitored by growth in the Algem HT24 photobioreactor. The lysis times for all these conditions were calculated as explained earlier and the results were plotted as shown in Figure 5.

In the graph, a linear relationship is evident between the different conditions, with an R value very close to 1, suggesting a good fit with the data. Using the equation from the trendline, the culture crash time can be determined for any given MOI. With those values, it is possible to calculate the order of magnitude of pfu/cell and, again, the result was comparable with the previous one of 10^5^ pfu/cell, increasing the confidence that the calculated value is a good approximation, because it is supported by multiple MOI conditions and multiple repeats.

This result suggests that the CviKI virus might have a higher binding affinity to cell debris compared to the PBCV-1 virus and shows that this tool could enable a new way of estimating a more accurate pfu released per cell measurement for all virus isolates.

### 3.5. Investigation of the Presence of Signalling Molecules

Another approach for which this system could be used is the evaluation of the presence of possible quorum-sensing signalling molecules smaller than 10 kDa. Suggestions of chemotaxis of *Paramecium bursaria* towards Chloroviruses are reported in the literature [24]. Another example of signalling molecules is the production of viral-specific glycosphingolipid (vGSL) in the coccolithovirus–*Emiliania huxleyi* system [25]. Therefore, we decided to investigate if signalling molecules from cultures infected at different MOIs, could have an impact on infected cells’ culture crash time.

In this instance, we investigated whether the infected cells were releasing signalling molecules, the presence of which may become apparent following infection with different MOIs. We theorised that the release of signalling molecules could have been related to the ratio between the number of cells and the number of viruses in the culture, which corresponds to the MOI.

**Figure 6:**
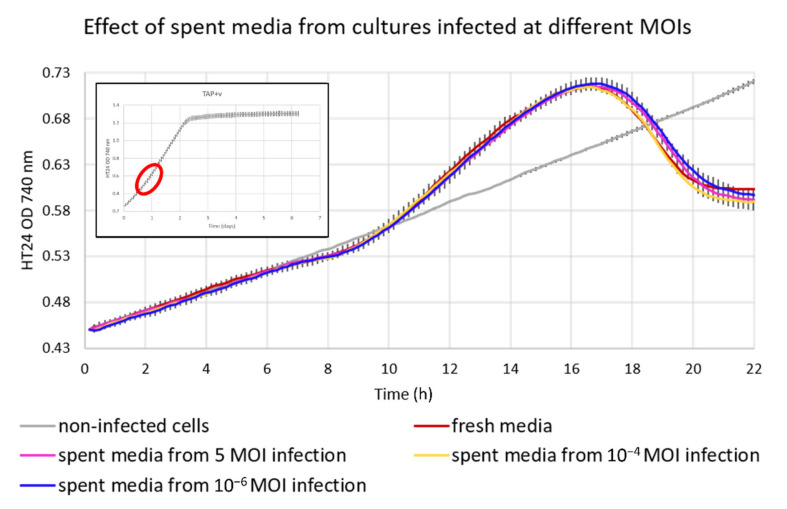
Infection of *Chlorella variabilis* cultures at 10^−4^ MOI with CviKI virus isolate and spent media from cultures infected at different MOIs, including a noninfected control. Cells were resuspended in fresh TAP medium for the no signalling molecules condition and with filtered spent medium for the other conditions as explained in the Materials and Methods section. The values are the average of 3 biological replicates with standard deviations. The inset graph from Figure 1 on the top left corner is used to highlight the scale of the closeup graph compared to the full growth of the uninfected organism, underlining that these are the fine-scale data focused on the initial part of the exponential growth.

The cultures were infected at 10^−4^ MOI and we included a noninfected control. We compared four conditions: cells resuspended in fresh medium, cells resuspended in spent medium collected from a culture infected with an MOI of 5, cells resuspended in spent medium collected from a culture infected with an MOI of 10^−4^, and cells resuspended in spent medium collected from a culture infected with an MOI of 10^−6^. The spent medium from all the different conditions was filtered twice. The first filtration eliminated the debris and the second stopped virus particles and any other molecule bigger than 10 kDa. The filtrate should, thus, contain any signalling molecules present smaller than 10 kDa. The cells were resuspended in the filtered spent media prior to a new infection to verify whether the lytic cycle of the virus would change in those conditions and to confirm or disprove the presence of signalling molecules. The average growth profiles of the resultant cultures with calculated standard deviations are shown (Figure 6).

Intriguingly, in all infected states, a minor decrease in OD was observed around 8 h p.i., probably due to the first round of cell lysis by the infected cells, which were about 1% of the total cell number, releasing virus particles that infected the remaining portion of noninfected cells in the culture. More surprisingly, the decrease in OD was subsequently followed by an increase in OD above that of healthy uninfected cultures for 7–8 h prior to a rapid decrease. The reason for this could be the accumulation of viral DNA and assembled virus particles inside the cells prior to cellular lysis, which could result in a change in cell/particle volume that is detected as an increased OD. There was no significant increase in cell culture density during the increase in the OD.

**Figure 7:**
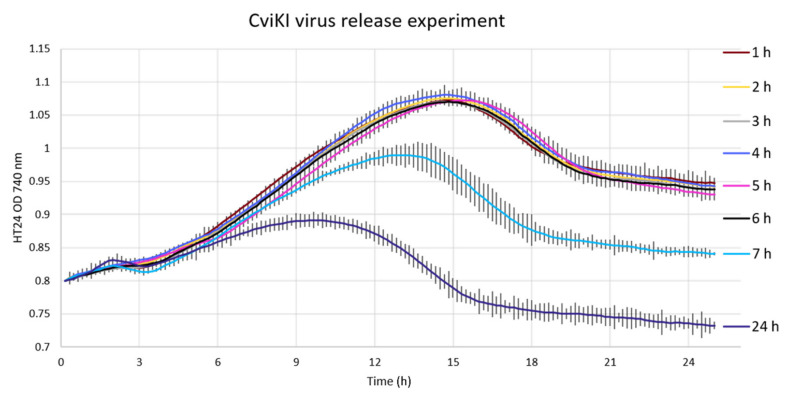
Infection of *Chlorella variabilis* cultures with filtered spent medium from previously infected cultures at 5 MOI with CviKI virus isolate sampled every hour up to 7 h p.i. and at 24 h p.i. The values are the average of 3 biological replicas with standard deviations.

This subtle pattern, which would most likely be missed without the automated nature of our sampling regime, was only observed in cultures infected at an MOI lower than 1, and it is also shown in Figure 7, but not present in Figure 2 with cultures infected at saturating MOI. There was no significant difference between the conditions implying that neither the infected cells nor the viruses are associated with quorum-sensing signalling molecules smaller than 10 kDa in any of the conditions tested.

### 3.6. Verification of the Virus Release Method after Infection

Another accessible application of the experimental approach described herein is the verification of the virus release process during infection. The three possibilities are:

1. By cell lysis, as already reported for this family of viruses [26], that is a sudden and large release of the virus particles [27];
2. By budding, which is a slower but controlled (constant or otherwise) release of virus particles [28,29];
3. A combination of both.

As Figure 7 clearly shows, the complete removal of all virus particles, even after five sequential washes, proved challenging; ultimately, every culture was observed to crash.

However, there was a marked difference between the infection dynamics observed from media taken 7 and 24 h p.i. Whilst 0–6 h all generated identical lytic curves, suggesting an almost identical amount of low-level crossover contaminating virus particles, cultures infected with media from 7 and 24 h p.i. produced a more rapid decrease in OD, with discernible profiles. At the 7 h time point, there was no evidence of lysis in the sampled cultures; it is possible that, with centrifugation, a few weaker cells with a damaged cell wall released infective virus particles.

After 24 h p.i., the cells were completely lysed and the virus titre was the highest, as suggested by the shorter lysis profile in Figure 7. This result confirms that the virus release method is via cell lysis because, if it would have been by budding, we would have expected to see a slow but constant decrease in culture crash time and not a sudden decrease as shown after 7 h p.i..

## 4. Discussion

The precise study of microalgae–virus infection dynamics is difficult to undertake due to long and variable infection cycles that can traverse light–dark cycles, requiring prolonged and intensive sampling regimes. Every sampling intervention potentially perturbs the environmental conditions and host–virus dynamic, introducing uncontrollable variation and, ultimately, decreasing precision and confidence in results.

In this study, we monitored infected cultures continuously (every 10 min) without interruption or intervention, while agitation, light intensity, and temperature were strictly controlled. The control and precision offered by our approach helped us to achieve high reproducibility, enabling us to visualise the subtle yet consistent differences between multiple virus isolates or of the same virus isolate under different conditions.

In this instance, we investigated the virus cycle of six different isolates and determined that each had different lysis times and OD profiles following infection (i.e., phenotype), identifying PBCV-1 as the fastest virus and MA-1D as the slowest virus with regard to completing a lytic cycle under the defined condition. We then determined the pfu/cell released and showed that the established approach could be highly valuable, especially for viruses with higher binding capacity to cell debris for which the plaque assay of the supernatant of lysed cells will underestimate and under-represent the true viral titre value. Through our new approach we identified that CviKI virus has the smallest ratio between the culture crash time at saturating MOI and at lower MOI when compared with the other isolates. We explored the potential biological reasons behind this observation as we calculated the order of magnitude of pfu/cell using the culture burst times at different MOIs.

Host–virus dynamics can be complicated by the presence of signalling molecules, as has been demonstrated in the coccolithovirus–*Emiliania huxleyi* system [25]. To this end, we investigated the possibility of a similar phenomenon occurring in the *Chlorella*–chlorovirus system. Our results provide strong evidence that no such signalling events occur in this system.

Perhaps the most clear-cut observation that we made related to the release of viral particles following infection was assessed through the development of a method to verify the mechanism, which was either at cell burst or by budding or a combination of the two. With this experiment, we confirmed previously reported findings for Chloroviruses [26], virus release initiating 6 h p.i. and being well established 7 h p.i. Crucially, early release before 6 h was not found to be a feature of the infection cycle.

Our data support a conclusion that, for CviKI, the infective virus particles released upon burst were strongly bound to cell debris, which would include cell wall and cell membrane components, as five sequential washes were not enough to eliminate all the infective virus particles bound to the cell debris. Indeed, our results indicate that, even right after the wash, there were still some virus particles in the medium that infected and lysed the cells (Figure 7). This observed phenomenon probably helps maintain the virus titre in environmental samples, explaining why they have just small fluctuations over the year [30]. The implications for the virus shunt and biogeochemical cycling of this observation are unknown but it could mean an underestimation of the viral component to the particulate organic matter (POM) due to the measurement of virus in just the dissolved organic matter (DOM) [31]. Based on what has been calculated herein, the number of infective CviKI viruses in the POM could be 100 times higher than the number of infective CviKI in the DOM.

All cultures exposed to spent media collected from a culture displaying a synchronised infection (washed five times to remove all unbound virus immediately following MOI 5 infection) lysed eventually (Figure 7), indicating a low level of transient or reversible binding of viruses to *Chlorella* cells. This means that, when we perform virus titration analysis in environmental samples, we are quantifying the fraction of the virus that is free or unbound to cell or cell debris. Therefore, when we infect a culture with lower MOI, the pfu released per cells could be greater than what can be measured, explaining why the virus isolate is perceived to have a shorter lytic cycle at lower MOI.

This conclusion does not hold for all the chlorovirus isolates investigated. For example, PBCV-1 at lower MOI lysed all the cells within the expected time windows, suggesting that there are relative differences in binding affinities to cell wall components between the different virus isolates. It is reported that chlorovirus stocks were more stable when stored with cell debris compared to filtration prior to storage [32], suggesting that storage in the presence of cell debris post-lysis may act to stabilise released viral particles from degradation and inactivation. We believe that storing chlorovirus particles with cell debris acts to not only stabilise them, but, due to a release over time in storage of those particles that are initially bound to debris and, therefore, un-infective, there is a greater stability of average pfu/sample over time. Particles that are initially un-infective within a given sample due to being bound to cell debris are released over time and become infective, replacing free viral particles that naturally deteriorate over prolonged storage. It is the relative on–off rate of the viral particle binding to cell wall components that could underpin the difference in behaviours observed across various viral isolates. It seems that the ratio between the free virus in the supernatant and the bound virus to cell wall/cell debris is constant, with an increase in both when adding more virus to the culture by cell burst, suggesting multiple binding sites on the *Chlorella* cell wall [32,33].

As we have shown, the Algem HT24 and Algem PRO are powerful tools for accurate and continuous monitoring of virus–host interactions. Potentially, other lab-scale photobioreactors that incorporate non-invasive continuous monitoring and precision control of parameters necessary to maintain defined growth conditions could also be used to follow microalgae viral–host interactions. Here, we gained insight on viral lytic cycles, mechanism of infection, stability, mechanism of virus release, quantification of pfu released per cell, and possible environmental impacts. In this instance, the combination of the larger-precision lab-scale photobioreactor (Algem PRO) affording accurate control of multiple variables with the higher throughput system (Algem HT24) enabled a deeper understanding of viral–host biology to be gained than could have been achieved through the use of one system alone. The approach and tools validated herein could be generally applied to any algal–viral system, enabling investigation into areas including but not limited to viral coinfection mechanisms, host/virus evolution, population/community dynamics, and, potentially, the impact of other microorganisms, including bacteria and predators, such as *Paramecium*, on virus/host systems.

## Figures and Tables

**Table 1:** Calculated average growth rates of *Chlorella variabilis* expressed in average change in OD value per day ± standard deviation and measured DW in g/L expressed as average ± standard deviation measured at day 6.24. All experiments were carried out with 3 biological replicates.

Medium	Average Growth Rate (OD 740 nm Increase/Day)	Average DW Measured at Day 6.24 (g/L)
MBBM	0.112 ± 0.010	0.42 ± 0.02
HSM+v + glucose	0.162 ± 0.008	0.74 ± 0.03
HSM+v + glycerol	0.148 ± 0.009	0.73 ± 0.03
HSM+v	0.131 ± 0.007	0.34 ± 0.02
TAP (ammonium)	0.499 ± 0.025	1.37 ± 0.06
TAP+v (ammonium)	0.499 ± 0.025	1.53 ± 0.05
TAP+v (urea)	0.065 ± 0.004	0.43 ± 0.02
TAP+v (nitrate)	0.008 ± 0.001	Too low

## Data Availability

The research data supporting this publication are provided within this paper.
